# Rehabilitation of Total Knee Arthroplasty by Integrating Conjoint Isometric Myodynamia and Real‐Time Rotation Sensing System

**DOI:** 10.1002/advs.202105219

**Published:** 2022-01-17

**Authors:** Jianzhe Luo, Yusheng Li, Miao He, Ziming Wang, Chengyu Li, Di Liu, Jie An, Wenqing Xie, Yuqiong He, Wenfeng Xiao, Zhou Li, Zhong Lin Wang, Wei Tang

**Affiliations:** ^1^ CAS Center for Excellence in Nanoscience Beijing Key Laboratory of Micro‐nano Energy and Sensor Beijing Institute of Nanoenergy and Nanosystems Chinese Academy of Sciences Beijing 101400 P. R. China; ^2^ School of Nanoscience and Technology University of Chinese Academy of Sciences Beijing 100049 P. R. China; ^3^ Department of Orthopedics Xiangya Hospital Central South University Changsha 410008 P. R. China; ^4^ National Clinical Research Center for Geriatric Disorders Xiangya Hospital Central South University Changsha 410008 P. R. China; ^5^ School of Material Science and Engineering Georgia Institute of Technology Atlanta GA 30332‐0245 USA; ^6^ CUSPEA Institute of Technology Wenzhou 325024 P. R. China; ^7^ Institute of Applied Nanotechnology Jiaxing 314031 P. R. China

**Keywords:** personalized healthcare, rehabilitation, self‐powered sensors, total knee arthroplasty, triboelectric nanogenerators

## Abstract

As the world population structure has already exhibited an inevitable trend of aging, technical advances that can provide better eldercare are highly desired. Knee osteoarthritis, one of the most common age‐associated diseases, can be effectively treated via total knee arthroplasty (TKA). However, patients are suffering from the recovery process due to inconvenience in post‐hospital treatment. Here, a portable, modular, and wearable brace for self‐assessment of TKA patients’ rehabilitation is reported. This system mainly consists of a force transducer for isometric muscle strength measurement and an active angle sensor for knee bending detection. Clinical experiments on TKA patients demonstrate the feasibility and significance of the system. Specifically, via brace‐based personalized healthcare, the TKA patients’ rehabilitation process is quantified in terms of myodynamia, and a definite rehabilitation enhancement is obtained. Additionally, new indicators, that is, isometric muscle test score, for evaluating TKA rehabilitation are proposed. It is anticipated that, as the cloud database is employed and more rehabilitation data are collected in the near future, the brace system can not only facilitate rehabilitations of TKA patients, but also improve life quality for geriatric patients and open a new space for remote artificial intelligence medical engineering.

## Graphical abstract

A portable, modular, and wearable brace for TKA patients’ rehabilitation is reported. It consists of a force transducer for isometric muscle strength measurement and an active angle sensor for knee bending detection. And it quantifying the TKA patients’ rehabilications and improve the rehabilication rate.

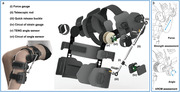

## 1 Introduction

The number of people over 60 years will outnumber young adults during the next ten years.^[^ ^1^ ^]^ Although human live longer, unfortunately, many do not live in good health later in life due to several diseases.^[^ ^2^ ^]^ Diseases associated with aging, such as joint‐degenerative diseases (osteoarthritis, OA), are prevalent among old people, reducing life quality not only for the elders but also for their guardians.^[^ ^3^ ^]^

Osteoarthritis (OA), a joint degenerative disease characterized by articular cartilage degeneration and secondary bone hyperplasia, is mainly manifesting as recurrent joint pain and movement disorders.^[^ ^4^, ^5^ ^]^ It is prevalent in middle‐aged and elderly people.^[^ ^6^, ^7^ ^]^ According to a survey conducted by the World Health Organization in 2015, the prevalence of symptomatic OA in men and women over 60 years is 18.0% and 9.6%, respectively.^[^ ^8^ ^]^ Among the patients, 80% have limited mobility and 25% of patients cannot do daily activities independently. Apart from inconvenience, the treatment is expensive too. Research statistics indicate that the cost of treating OA has accounted for 1.0 to 2.5% of the gross national product GDP of developed countries.^[^ ^9^ ^]^ Knee osteoarthritis (KOA),^[^ ^10^, ^11^, ^12^ ^]^ the most common OA, not only causes ache and dysfunction,^[^ ^13^, ^14^ ^]^ but also induces psychosocial anxiety, helplessness, depression, and social disorders.^[^ ^15^, ^16^ ^]^ For advanced degenerative KOA, total knee arthroplasty (TKA) is now generally regarded as a safe and effective treatment.^[^ ^17^, ^18^ ^]^ The annual number of total knee replacement in the United States is predicted to increase progressively from 1 065 000 to 1 921 000.^[^ ^19^ ^]^ Although millions of surgeries are completed, after surgery, these individuals are always plagued with quadriceps muscle impairments and functional limitations,^[^ ^20^ ^]^ which might be a combination of muscle atrophy and neuromuscular activation deficits.^[^ ^21^, ^22^ ^]^ To avoid side effect and ensure desirable long‐term functional gains, which would influence the life quality of individuals, chronic quadriceps muscle impairments should be tackled adequately. Therefore, during the recuperative period, longitudinal and accurate rehabilitation assessment, composed of quantitative medical indicators, is indispensable and critical for valid treatment.^[^ ^23^, ^24^ ^]^

For now, two typical muscle testing systems are developed, that is, isokinetic muscle strength assessment and training system,^[^ ^25^, ^26^, ^27^ ^]^ and manual muscle testing systems.^[^ ^28^, ^29^ ^]^ These two systems can provide sufficient medical examination for doctors during hospitalization, but they are either bulky or requires doctors’ operation experience. Therefore, they cannot be widely applied to postoperative and long‐term rehabilitation evaluation of patients. To achieve postoperative and longitudinal rehabilitation monitoring of patients, wearable technologies, mainly based on inertial measurement units (IMUs) with accelerometers and gyroscopes, are proposed.^[^ ^30^, ^31^, ^32^, ^33^, ^34^ ^]^ IMUs are able to measure the knee bending motions, but cannot assess myodynamia. Additionally, the IMUs measure indirectly, requiring complicated body parameters for post computational modeling to calculate out the motions, and extra corrections, from time to time, to reduce the misalignment error that grows as a function of time.^[^ ^35^ ^]^ Thus, new wearable long‐term monitoring technology is in demand.

To date, many patients choose to use knee brace. As global knee braces market report states, the worldwide quantity demand dramatically increases in the recent, and the market size is expected to reach $1.9 billion by 2025.^[^ ^36^ ^]^ Knee brace, a device for replacing cumbersome and airtight plaster in post‐hospital protection, is widely used in hospital and domestic environment among patients with tendonitis, OA, and other injuries,^[^ ^37^, ^38^, ^39^ ^]^ making it an ideal carrier of personalized intelligent healthcare^[^ ^40^ ^]^ and conductive to the recuperation of orthopedic patients.^[^ ^41^ ^]^ Triboelectric nanogenerator (TENG), first invented by Zhong Lin Wang et al in 2012, is a promising technology that might facilitate IoT,^[^ ^42^, ^43^, ^44^, ^45^ ^]^ artificial intelligence (AI),^[^ ^46^, ^47^, ^48^, ^49^, ^50^ ^]^ and personalized medicine^[^ ^51^, ^52^, ^53^, ^54^, ^55^ ^]^ via self‐powered motion sensors. Compared to traditional sensors, TENG based angle sensors have the advantages of light weight, wide selection of materials, and self‐powered ability.^[^ ^56^, ^57^ ^]^ In our previous work, a self‐powered angle sensor based on TENG is reported and embedded in a knee brace for bending detection.^[^ ^58^ ^]^ However, only information of movement detection is not sufficient for rehabilitation assessment.

Here, we report a portable, modular, and wearable brace for self‐assessment of TKA patients’ rehabilitation. A strength measurement module is characterized via a standard torque assessment platform. The relationship between the loaded torque and the muscle forces measured by our strength module of the telescopic rods is recorded. As for the angle measurement module, liquid lubrication^[^ ^59^ ^]^ and sponge buffering are introduced for ensuring its long‐term working stability. Clinical experiments are performed, and demonstrate that the brace realizes the quantification of the TKA patient's recovery process, showing a gradual increasing trendy of the myodynamia after surgery. Moreover, facilitated by the conjoint rotation sensing module and cellphone application program, we can assess the daily activities of patients after surgery, and give out home training instructions. It is found that, the intervention group shows an apparent enhancement both in muscle force and joint bending range, compared to the control group. Furthermore, we establish a new rehabilitative indicator, isometric muscle test score (IMTS), for quantitatively evaluating TKA rehabilitation. We anticipate that, as the cloud database is employed and more rehabilitation data are collected, the brace system could not only facilitate rehabilitations of TKA patients, but also improve life quality for geriatric patients, and even open a new space for remote AI medical.

## 2 Results

### 2.1. Overall Flow

**Figure 1:**
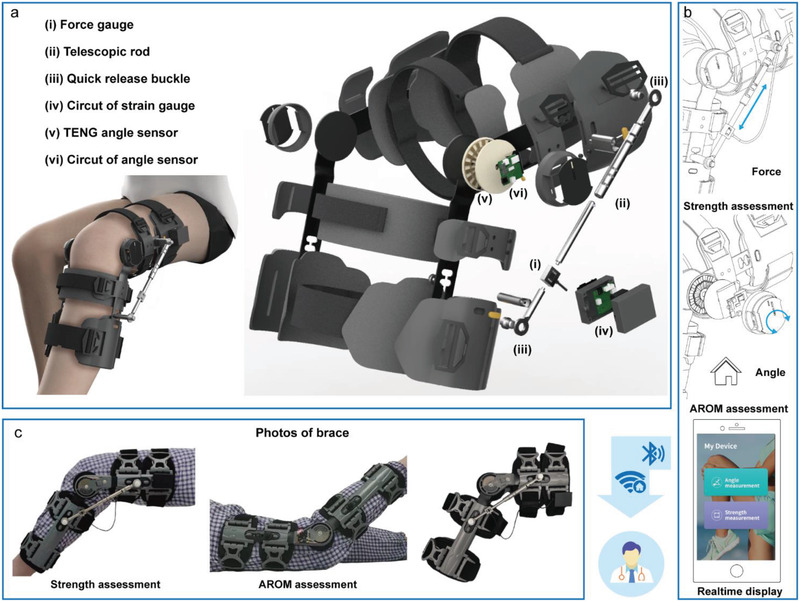
Schematic illustration of the rehabilitation brace system. a) A perspective view of a brace with modular device for measuring isometric myodynamia and joint ROM (range of motion). The brace system includes i) a force gauge; ii) telescopic rod corresponding to brace bending angle of 30°, 50°, 60°, 90°; iii) quick release buckle for telescopic rod fixation and disassemble; iv) data processing circuit of force gauge for isometric muscle measurement; v) active angle sensor with resolution of 1°; vi) data processing circuit of angle sensor for joint movement. b) Sketches of data collecting and transmitting. c) Measurement performing.

We developed two sensing modules that can be mounted onto current braces (**Figure** **1**), including isometric myodynamia measurement (real‐time force analysis) and active range of joint movement measurement (real‐time angle analysis). Figure 1a depicts the donning and exploded views of the brace system, which mainly consists of a force gauge, a telescopic rod, and an active angle sensor (the total weight of them is merely 71.0 g, see Figure S3, Supporting Information). Via the brace system, force and angle information can be recorded and displayed on the cellphone, accessible to patients and doctors, benefiting for rehabilitation monitoring (Figure 1b). Figure 1c shows the photo of the brace and how to perform the measurements.

### 2.2. Isometric Myodynamia Measurement Module

**Figure 2:**
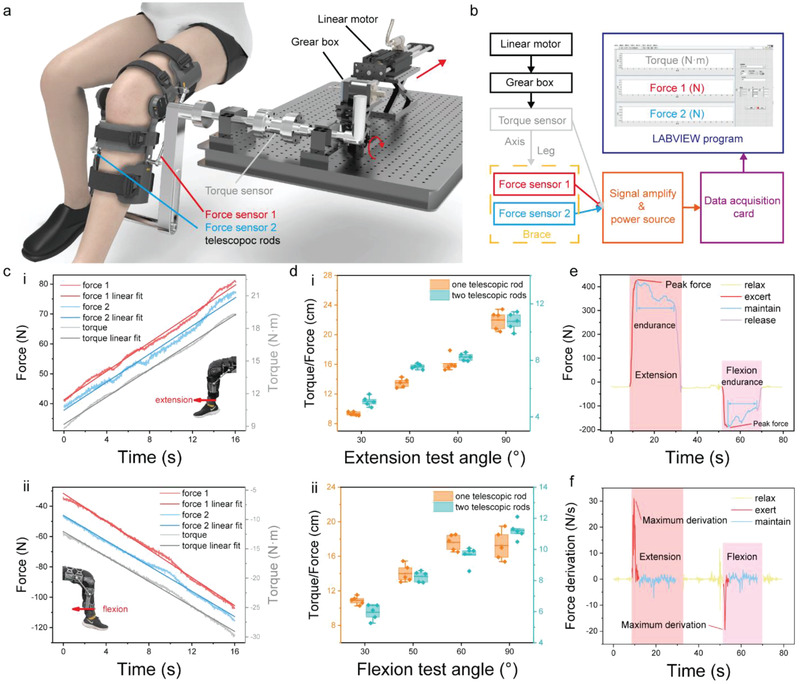
Characterization of the strength measurement module. Relationship between torque and force is explored through a precise concentric transmission. a) Schematic of the configuration of a transmission system for calibrating the strength module. The direction of torque transmission is shown by red arrow. b) Flow chart demonstrating the key steps of the signal acquisition and visualization. Once the linear motor slides, rotations of shaft and arm will be triggered, respectively. At the meanwhile, two force gauges will detect the force applied on the brace through leg. c) Measured relationship among real‐time extension and flexion forces and torque in the test system. d) Calculated torque/force ratios at 30°, 50°, 60°, 90° under one‐telescopic‐rod condition and two‐telescopic‐rod condition. e,f) Medical convalescent information derived from acquired isometric muscle strength test.

Since traditional muscle function analysis is normally based on torque, in this proof‐of‐concept research, we explore the relationship between torque and force via a standardized test platform, as shown in **Figure** **2**a. The standardized test platform is aimed to gain mechanical response of the brace system under linear torque stimulus, which is provided by a linear motor and a gear box. The procedure of the mechanical characterization is shown in Figure 2a,b. To verify the positive/negative correlation between torque and tension, one participant, who wear the brace, is instructed to sit right beside the platform and keep lower limb muscles relaxed during the test. We assure that the knee brace is coaxial with the transmission and no residual torque left. Under this configuration, the torque measured by the torque sensor is the torque applied to the brace. Furthermore, a three‐channel electrical acquisition and analysis system is developed to display values of torque and forces in the experiment. In the isometric extension simulation experiment, extension force generated by muscle extension is obtained from force transducers on the telescopic rods, while the applied torque is detected by the torque sensor. The positive/negative correlation between force and toque in extension/flexion simulation experiment is shown in Figure 2c. The red, blue, and grey lines represent the indications of force transducer 1 (at the outer side of brace), force transducer 2 (at the inner side of brace), and torque transducer (installed on the standard characterize platform), respectively. Notably, transducer 2 and the inner‐side rod are normally replaced with an angle limiter in daily use. Herein, they are assembled for the contrast experiment. An apparent linear relationship between the torque and the force measured by transducer 1,2 can be obtained from Figure 2c. The ratio of torque to force under various extension and flexion angles are shown in Figure 2d. The trend is consistent with the theoretical analysis (see in Note S1 and Figures S1 and S2, Supporting Information). It also indicates that the results obtained from one telescopic rod corresponds well with that from two telescopic rods, therefore, one telescopic rod is sufficient for myodynamia strength assessment. One testing curve of myodynamia is illustrated in Figure 2e,f, from which, we can obtain the dynamic myodynamia information, such as peak force, endurance time, maximum derivation of force, representing maximum muscle strength, muscle power, and endurance in medical application, respectively.

### 2.3. Active Rotation Sensing Module

**Figure 3:**
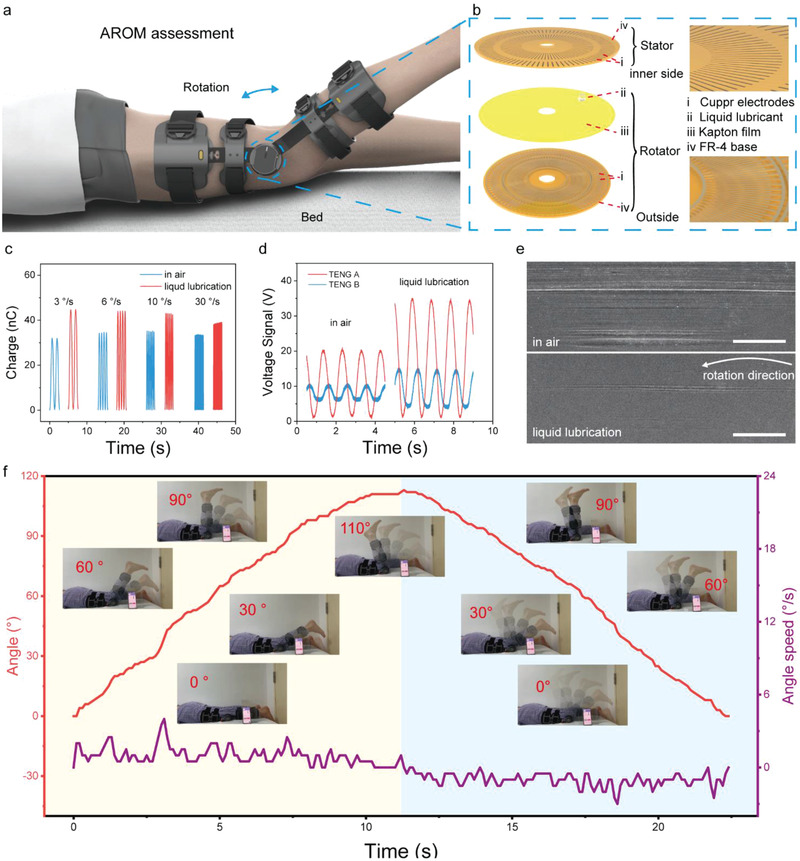
The response of brace angle measurement module. Schematic illustration of a) active range of movement test and b) TENG liquid‐enhanced angle sensor with interface liquid enhancement c) Transferred charge of a TENG angle sensor in air and with interface liquid lubrication. d) Open‐circuit voltage of TENG angle sensor in air and with interface liquid lubrication. e) SEM images results of Kapton film surface after 100 000 cycles in air and with interface liquid lubrication (scale bar, 100 µm). f) The real‐time angle signals, angle speed, and photos during the active range of movement test.

The active range of movement measurement, supported by the brace, is demonstrated by **Figure** **3**a. Exploded view of the active angle sensor is shown in Figure 3b. The sensor (60 mm in diameter) mainly consists of FR‐4 substrates (1 mm in thickness), grid electrodes (≈0.1 mm in width), Kapton film (40 µm in thickness), and liquid lubricant (20 µL in volume). Working principle can be found in Figure S7, Supporting Information, and our previous work.^[^ ^58^ ^]^ To improve the stability and reliability of the sensors, we investigated the effect of different lubricants on the signal (see in Figure S8, Supporting Information). The introduction of the lubricant (Figure 3d,e), here preferably squalene, enhances the voltage signals that generated by two sets of TENGs (Figure 3c,d), which is beneficial for the rotation angle detection. More importantly, the durability of the TENG angle sensors is significantly improved by the liquid lubrication. Compared with air lubricant, liquid modification can increase the durability of TENG during 100 000 continuous cycles of rotation regarding transfer charge signals.^[^ ^59^ ^]^ Additionally, liquid lubrication significantly minimized Kapton film surface scratches as shown in SEM images of Figure 3e.

For the purpose of detecting real‐time angular information, we developed a circuit to acquire the signal generated by TENG angle sensors and then, transmitted to a cellphone terminal via Bluetooth and display the angle information through the application program (APP) on the cellphone. Detail information about the APP is shown in Figure S8, Supporting Information. Angle sensing with a resolution of 1° is achieved. Demonstration of the real‐time angle sensing is plotted in Figure 3f. Original derived signals is angles, while derivation of angle, rotation speed is the blue line. In the measurement, the patient bends his knee from 0° to 110° and move back to 0°.

### 2.4. Quantification of the TKA Patient's Recovery Process

**Figure 4:**
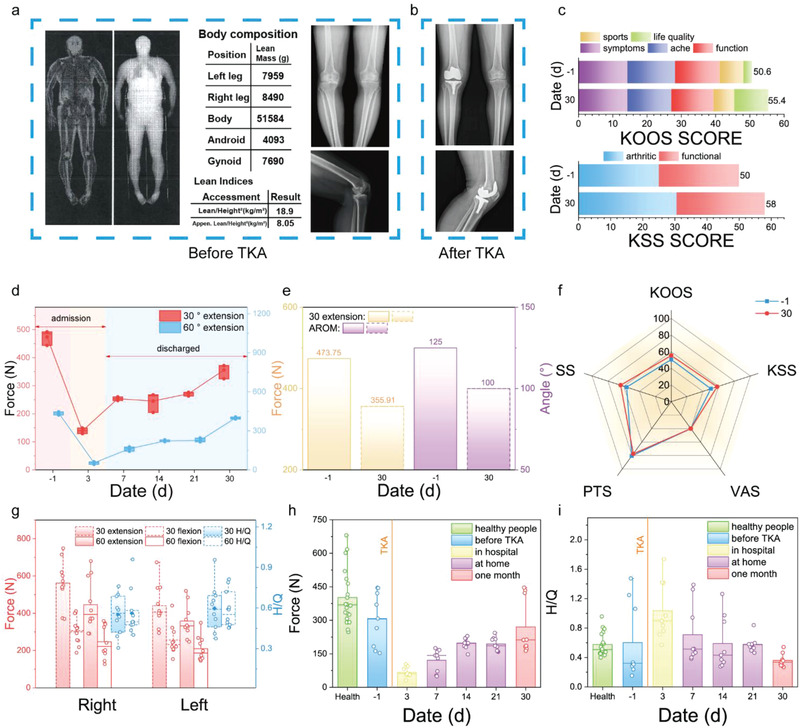
Clinical analysis of TKA patients’ rehabilitation through brace system. a) Dual energy X‐ray body composition analysis images and data; as well as X‐ray images of a patient before TKA. b) X‐ray images of the patient after TKA. c) Composition of KOOS and KSS scores before and after TKA: the day performing TKA is regarded as 0, −1 represents the day before TKA and 30 represents the 30th day after TKA. d) Isometric muscle tests of the patient during one month (extension peak force) e) 30° extension peak force and active range of motion of the patient before TKA and one month after TKA. f) Rehabilitation indicators comparison between pre‐operation and post‐operation. g) Isometric muscle tests data of 10 healthy participants. A group of patients’ 60° isometric muscle tests in one‐month scale: h) Extension peak force and i) hamstring to quadriceps (H/Q) ratio.

Pre‐operative information, dual energy X‐ray body composition analysis and X‐ray images, of a patient is shown in **Figure** **4**a. After the TKA, X‐ray images (Figure 4b) of the patient is taken to validate the success of the procedure. It is found that the surgery is successful and body composition information is shown in the chart of Figure 4a. Apart from these messages, other medical indicators, including knee injury and osteoarthritis outcome score (KOOS), American knee society knee score (KSS), visual analogue scale (VAS), psychological test score (PTS), and step speed (SS) of the patient before and after the operation are recorded. Figure 4c presents the patient's KOOS and KSS score composition (−1 and 30 represent the day before and one month after surgery, respectively). To increase comparability among all these scores, we rescaled the total number of scores to 100 without changing the composition proportions of the scores. To a certain extent, the patient seems to have recovered partially after the first month of operation, although the rehabilitation is not completed. Meanwhile, we applied the myodynamia measurement module to the patient for one‐month longitudinal monitoring, and quantitatively determined the rehabilitation level of muscular strength. From the 30° and 60° extension results (Figure 4d), we can find that muscle strength (force) of the patient did increase gradually after surgery, yet fail to reach to the preoperative status on the 30th day. Other traditional medical indicators, KOOS, KSS, VAS, PTS, and SS, are shown in Figure 4f. Variation of these indicators in percentage are 9.48%, 16%, 0%, −3.80%, and 13.54%, respectively. However, 30° extension force and ROM declined by 24.87% and 28%, respectively (Figure 4e). To establish a reference myodynamia library measured by the brace, 10 healthy candidates (5 males and 5 females) participated the measurement and their lower limp muscle strength data at two typical angles were collected. In Figure 4g, relevant clinical data, including peak force and hamstring to quadriceps (H/Q) ratio, are extracted from the isometric muscle tests which repeated at least three times for a participant at one angle. The average right/left limb peak forces of isometric myodynamia measurements, which are manipulated under 30° extension, 30° flexion, 60° extension, and 60° flexion, are 562.56/440.81 N, 299.74/256.45 N, 446.35/357.81 N, and 247.25/208.86 N, respectively (Figure 4g red part). The average right/left leg H/Q ratios, tested under 30° and 60°, are 0.55/0.60 and 0.56/0.59, respectively (Figure 4g blue part). Additionally, isometric myodynamia data of patients (three) are investigated in one‐month duration, and plotted in Figure 4h,i and Figure S10, Supporting Information. Here, the trend of isometric muscle strength of patients was consistent with the tendency of the patient mentioned above, showing a decline on the third postoperative day as well as a slow post‐surgery increase, however, neither myodynamia nor H/Q ratio returned to preoperative status.

### 2.5. Rehabilitation Enhancement

**Figure 5:**
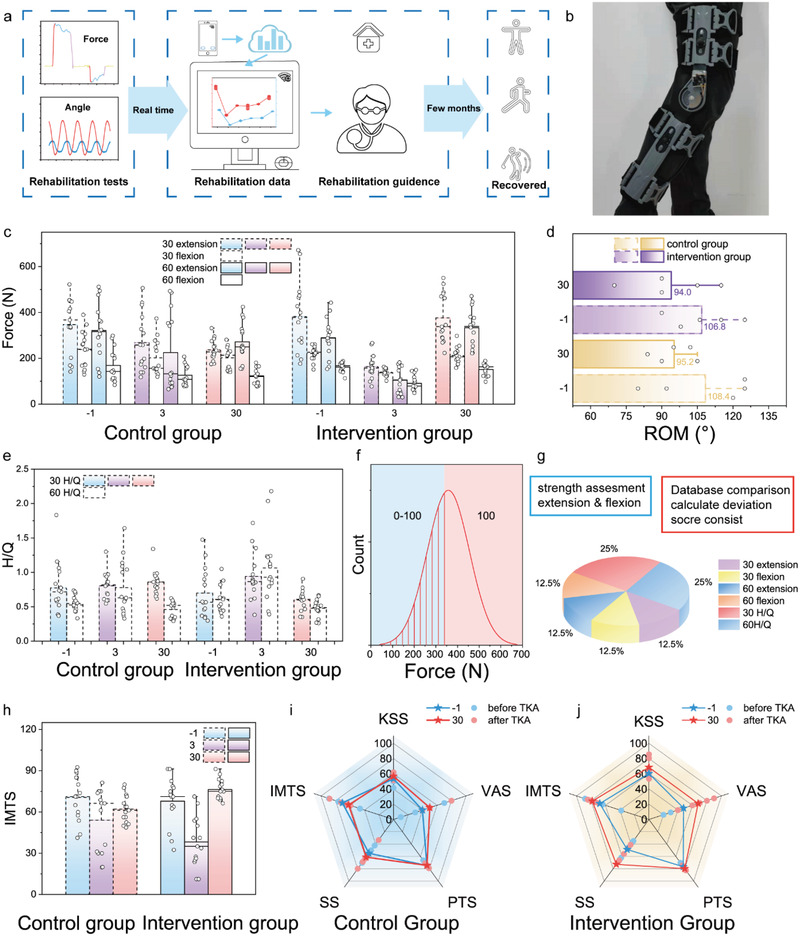
Brace‐based personalized medicine contributing to convalescent. a) Schematic images of brace system's effect on TKA patients over the rehabilitation period. b) Photo of exercise with brace. c) Myodynamia data of control and intervention group under 30 extensions, 30 flexions, 60 extensions, as well as 60 flexion isometric tests. d) ROM of controlled group and intervention group tested on day 1, day 3, and day 30. e,f) Normal distribution of force from 60 extension isometric test. g) Components of IMTS and score assignment principal. h) IMTS scores of controlled group and intervention group tested on day 1, 3 30. i,j) rehabilitation indicators of controlled group and intervention group.

Subsequently, two groups of patients (5 patients each group) were randomly selected for verifying the effects of the brace on the rehabilitation. Overall procedure of brace‐assisted rehabilitation healthcare is shown in **Figure** **5**a. The intervention group are monitored through the brace, as shown in Figure 5b. Therefore, their daily bending motions are able to be sent to the doctor, who will then give exercise suggestions back. The isometric muscle strength data of the intervention and control groups are shown in Figure 5c. Compared with the preoperative myodynamia results, the data on the third day of both the intervention and control groups dropped dramatically, but after one month, the recovery of intervention group, with brace intervention, was apparently enhanced compared to that of control group in terms of muscle strength. For instance, on average, the muscle strength measured under 30° extension was recovered to 68.35% compared to the preoperative status for the control group, whereas, that was recovered to above 98.41% for the intervention group. Figure 5d shows the data of patients’ knee bending ability before and after TKA in both groups, which is evaluated by ROM. And the ROM shows a reduction induced by the surgery on the 30th day. Specifically, the reduction was found to be lower in the intervention group (−11.99%) than that in the control group (−12.18%). Moreover, the intervention group also recuperated better regarding H/Q ratio in comparison to the control group, that is, intervention group's outcomes were closer to the normal human H/Q ratio. To improve the comparability of the measured isometric myodynamia data, we proposed an evaluation standard, named with IMTS, as follows (Figure 5f,g): the score is a weighted sum of isometric test scores of 30 extension, 30 flexion, 60 extension, 60 flexion, 30 H/Q, 60 H/Q; each test score is obtained through comparing the testing result with the normal distribution of healthy people's data; the percentage of each test score is illustrated in Figure 5g (detail scoring information see Note S2, Supporting Information). Subsequently, IMTSs of two groups before and after TKA is shown in Figure 5h. When compared to the presurgical IMSTs, the IMSTs of the control group reduced by 12.04% one month after operation, while the IMSTs of the intervention group rose by 15.36%. A new comprehensive evaluation standard in the form of radar graph, comprising of traditional medical indicators, and IMTS, are established and illustrated in Figure 5i,j (detail information see Table S2, Supporting Information). It can be found that the proposed standard shows a clear distinction between the two groups. Additionally, the intervention group’ rehabilitation is apparently enhanced. By summing up the scores of multiple indicators, of control and intervention group, the variations are found to be 3.77% and 21.90%, respectively.

## 3 Conclusion

Here, we report a portable, modular, and wearable brace for self‐assessment of TKA patients’ rehabilitation. The brace consists of a force transducer for isometric myodynamia measurement and an active angle sensor for knee bending detection. In addition, the force and angle resolutions are 0.01 N and 1°, respectively. Clinical experiments on TKA patients (*n* = 14) and healthy people (*n* = 10) demonstrate the system feasibility. Key advances are as follows: 1) the capability in quantifying the TKA patients’ rehabilitation process in terms of myodynamia; 2) the definite rehabilitation enhancement due to the conjoint myodynamia assessment and knee bending sensing; 3) new quantified standard indicators, that is, IMTS, for evaluating TKA rehabilitation is proposed based on the brace‐assisted approach.

Notably, patients’ rehabilitation data including myodynamia and knee bending activities can be recorded and further uploaded onto a cloud database for TKA rehabilitation. Doctors can expediently give out rehabilitation advices to patients on line, facilitating patients to reach ideal prognosis after surgery. Additionally, we anticipate that, combing the database with the machine‐learning analysis, personal rehabilitation advices can be generated directly, which will improve life quality for geriatric patients and even open a new space for AI medical consulting.

## 4 Experimental Section

### Fabrication of Telescopic Rods

To begin with, range of torque for human isometric rods was investigated and converted to relevant force on telescopic rods. Then proper positions on the brace for fixed points were selected which means the length of telescopic rods at different angles. Mechanical draws and 3D model were created based on upper principals. Traditional manufacturing processes, which included laser cutting, oxidation coating, and gridding, were applied in manipulating the telescopic rods. Here, the materials for the rods were high young's modules metal, preferably chosen aluminum and carbon fiber for lightweight.

### Fabrication of Standard Platform

The torque sensor with a range of 200 N was selected first, and the corresponding transmission structure, components of which were finished by machining and surface corrosion treatment, was designed according to this torque sensor.

### Synthesis of TENG Angle Sensors

The fabrication of the TENG based angle sensor was mainly based on the mature printed circuit board technology. Thus, schematic illustrations of SPAS's multilayer structures were necessary before the automatic production. An electronic structural design software named of Altium Designer 16 was used to depict the structural sketches. Detailed fabrication procedures are revealed as follows: The substrate chosen for both the rotator and stator was FR‐4 epoxy glass with a thickness of 1 mm. To transfer patterns of copper to this substrate, first it was covered with a complete copper sheet with a thickness of 50 µm on FR‐4 base through cold rolling craft followed by laminate a sensitive dry layer on the top of the copper sheet. After that, the sensitive layer was exposed to patterned UV light via photo tools, and the unexposed part of this layer was removed by the developing solution. Before the strip of the dry film layer, redundant copper was etched by ferric chloride solution and removed from the patterned copper. Finally, a Kapton film with thickness ≈50 µm was attached to the stator part to function as a triboelectric layer while a layer of gold was deposited to the surface of patterned copper of the rotator part to prevent copper from oxidation.

### Integration of ROM Module and Myodynamia Module

On the one hand, the TENG angle sensor was embedded between the friction subsets of the brace with squalene lubrication and sponge cushioning. On the other hand, after the fixed columns of the muscle module were mounted on the outside of the brace, the telescopic rod and other components were assembled subsequently on fixed columns which is depicted in Figure 1a.

### Statistical Analysis

Pre‐processing of data: isometric muscle test data was obtained by subtracting the maximum and minimum values from the baseline. Processing of IMST data is shown in Note S2, Supporting Information. Other data (such as transferred charge, short‐circuit current, open‐circuit, ROM, force, and torque) are presented without pre‐processing. Data presentation: mean ± SD; mean. Sample size (*n*): Figure 2d (5); Figure 4d (3); Figure 4g (15); Figure 4h (9); Figure 5c, e, h (30); Figure 5d (5). Software used for statistical analysis: Origin 2021.

### Ethics Oversight

All procedures in the tests in healthy individuals and TKA patients were in accordance with the experimental protocol approved by the Committee on the Use of Humans as Experimental Subjects of the Xiangya Hospital, Central South University (COUHES, no. 201 908 798). All participants were informed with written consent.

## Conflict of Interest

The authors declare no conflict of interest.

## Supporting information

Supporting Information

Supplemental Movie 1

Supplemental Movie 2

## Data Availability

The data that support the findings of this study are available from the corresponding author upon reasonable request.
