# Supplementary material for: Rehabilitation of Total Knee Arthroplasty by Integrating Conjoint Isometric Myodynamia and Real‐Time Rotation Sensing System
Source: Adv Sci (Weinh). 2022 Jan 17;9(8):2105219. doi: 10.1002/advs.202105219 (PMC8922106; doi:10.1002/advs.202105219)
Supplement: Supplementary file 1 — Supporting Information [file ADVS-9-2105219-s001.pdf]

ADVANCED SCIENCE
Open Access

Supporting Information

for *Adv. Sci.*, DOI 10.1002/advs.202105219

Rehabilitation of Total Knee Arthroplasty by Integrating Conjoint Isometric Myodynamia and Real-Time Rotation Sensing System

Jianzhe Luo, Yusheng Li, Miao He, Ziming Wang, Chengyu Li, Di Liu, Jie An, Wenqing Xie, Yuqiong He, Wenfeng Xiao, Zhou Li, Zhong Lin Wang* and Wei Tang**

© 2020 Wiley-VCH GmbH

ADVANCED SCIENCE
Open Access

Supporting Information

for *Adv. Sci.,* DOI: 10.1002/advs.202105219

Rehabilitation of Total Knee Arthroplasty by Integrating Conjoint Isometric Myodynamia and Real-Time Rotation Sensing System

Jianzhe Luo, Yusheng Li, Miao He, Ziming Wang, Chengyu Li, Di Liu, Jie An, Wenqing Xie, Yuqiong He, Wenfeng Xiao, Zhou Li, Zhong Lin Wang*, and Wei Tang**

Supporting Information

Rehabilitation of total knee arthroplasty by integrating conjoint isometric myodynamia and real-time rotation sensing system

Jianzhe Luo, Yusheng Li, Miao He, Ziming Wang, Chengyu Li, Di Liu, Jie An, Wenqing Xie, Yuqiong He, Wenfeng Xiao, Zhou Li*, Zhong Lin Wang*, Wei Tang*

Table of contents

Supporting Text 3

Note S1: Theoretical analysis of the isometric myodynamia measurement. 3

Note S2: Scoring principle of isometric myodynamia test score (IMTS). 3

Note S3: The reason for selecting TENG as an angle measurement module. 4

Supporting Tables 4

Table S1. Comparison summary of different systems for TKA patients 4

Table S2. Mean values of medical indicators for the control and experimental groups in Fig. 5i-j. 5

Table S3. Main features of commercial angle sensor and their comparison with TENG angle measurement module .. 5

Supporting Figures 6

Figure S1. Isometric myodynamia measurement sketches 6

Figure S2. Force analysis of the brace 7

Figure S3. Photos of sensors and telescopic rods 8

Figure S4. Interfaces of the APP 9

Figure S5. Circuit photos of strength and angle measurement modules 10

Figure S6. Images of the precise concentric transmission for brace mechanical characterization 11

Figure S7. Mechanism diagram of the TENG-based angle sensors 11

Figure S8. Powder and liquid lubrication for TENG-based angle sensors 12

Figure S9. Rotation simulation experiment 13

Figure S10. Longitudinal rehabilitation monitoring a group of patients via IMT 14

Figure S11. TENG output under different lubricant situations 15

Figure S12. The output performance of the TENG angle sensor in two places 15

Supporting Videos

Video S1. Isometric myodynamia measurement 16

Video S2. Active range of motion measurement 16

Supporting Text

Note S1. Theoretical analysis of the isometric myodynamia measurement for the force gauge and telescopic rod.

To begin with, we simplify the brace, as illustrated in Fig. S1a-b, and its triangular relationship is shown in Fig. S1c. Then, we approximated the brace fixed to the leg as Fig. S2a, which is because the thigh part of the brace is fixed to the thigh and the thigh is fixed to the chair or bed. Similarly, the thigh and calf parts of the support are approximated as fixed rods, and the support pivots, fixed ends A, B are considered as articulation points.

Take the knee extension test as an example, at rest, the force of the human leg on the support is gravity, and when the human leg is kicking, the force of the human leg on the support is simplified to F, which is perpendicular to the AC bar (Fig. S2b).

Point A, B, C are hinges, so in the case of not counting the weight of the rod, AB can be regarded as a two-force rod and the force is along the rod direction, as shown in Fig. S2c. When the leg is relaxed, the force measured by the force sensor is the pressure, and the force from the lower limb to the support is G_1. According to the principle of convergence of the three forces, the force on the AC rod can be found (Fig. S2d).

When solving for the torque W of the human leg on the support, we equate it to the force acting vertically on the AC rod at point A (the force along the AC rod has no effect on the force on the AB rod and can be ignored), and the same principle of convergence of the three forces can be used to find the force at point A (Fig. S2d).

Comparing the state of force in brace before and after the kicking, we can find:

$$Fa = \Delta F \tag{S1}$$

$$W = \Delta Fbsin\alpha \tag{S2}$$

Based on the previous geometric relationship, we get:

$$W = \Delta Fbsin(arccos\frac{b^2 + c^2 - a^2}{2bc}) \tag{S3}$$

To obtain the relationship between the moment/force ratio and the isometric muscle force test angle, it is transformed into:

$$W/\Delta F = bsin(arccos\frac{b^2 + c^2 - a^2}{2bc}) \tag{S4}$$

That is, the telescopic rod (b) shortens, the angle (α) becomes larger, the ratio increases, which is consistent with our Torque/Force ratio trend in Fig. 2d.

Note S2. Scoring principle of isometric myodynamia test score (IMTS).

Data of 10 healthy people are recorded for standard library (Fig. 4g). Then, we assume that the data for healthy individuals are normally distributed with the mean of the 10 individuals measured in

this trial. We then divided the normal distribution into eleven regions (scores 0-100), as shown in the fig. 5f, where scores above the mean are 100 and regions less than the mean are evenly divided into scores 0-90. The assignment of H/Q differs from that of force in a similar way, centered on the mean value and divided into 21 regions, with scores greater than twice the mean value being 0. Take 300 N (30 extension, female, right leg) and 0.8 (30 H/Q ratio, female, right leg) for examples, 300 N in the 50-score region (265 N – 318 N) and 0.8 in the 60-score region (0.77 – 0.83).

Note S3. The reason for selecting TENG as an angle measurement module.

First of all, TENG angle sensor is self-powered, which surpass other technologies like optoelectrical effect, Hall effect and an inertial measurement unit (IMU). Secondly, Electromagnetic angle sensor, optoelectrical angle sensor are bulkier, which making it difficult to be embedded in the brace. Thirdly, inertial measurement unit (IMU) would induce accumulation of errors and increase system complexity.

Supporting Tables

Name	Isometric testing and rehabilitation	Potable muscle strength tester	This work
Cost	+++	++	+
Accuracy	+++	+	++
Potability	+	+++	+++
Resolution	0.1 N·m; 1 °	0.1 N	0.01 N; 1 °
Therapist	Y	Y	N
Time	+++	+	+
Data availability	++	+	+++
Long-term monitoring for TKA	N	N	Y

Table S1. Comparison summary of different systems for TKA patients.
Comparison of different systems shows that our system is suitable for widely application of longitudinal monitoring in TKA patients' recuperation.

	Control Group			Experiment Group		
Name	-1	30	Increment	-1	30	Increment
KSS	53.5	56.8	6.17%	60.3	68.5	13.60%
VAS	40	50	25%	48	68	41.67%
PTS	74.69	74.59	-0.14%	76.63	80	4.39%
SS	54.93	61.31	11.61%	48.79	72.84	49.28%
IMTS	70.58	62.08	-12.04%	67.83	78.25	15.36%
SUM	293.71	304.78	3.77%	301.58	367.59	21.90%

Table S2. Mean values of medical indicators for the control and experimental groups in Figure 5i-j. Data are listed in blue for the day before surgery and in red for one month after surgery. Yellow font is the data of the experimental group that is larger than the control group.

Features	Mechanism	Power consumption	Weight	Thickness	System complexity	Limiting Resolution
Commercial sensors	Hall effect	0.5 W	100	53	Simple	3.6 degree
Commercial Sensors	IMU	0.1W	20	3	Complex	1 degree (Z)
TENG angle module	Triboelectric	Self-powered	11.1	1.87	Simple	1 degree

Table S3. Main features of commercial angle sensor and their comparison with TENG angle measurement module.

Supporting Figures

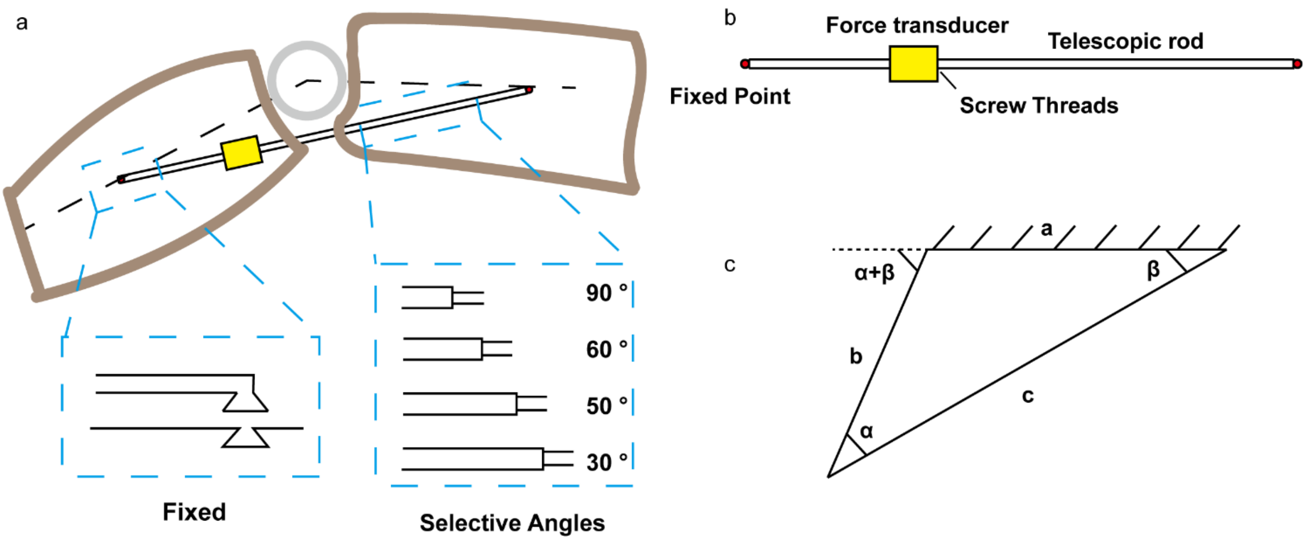

Figure S1. Isometric myodynamia measurement sketches. a) Sketch of brace and the principle of angle adjustment. **b)** Sketch of telescopic rod and force transducer. **c)** Equivalent geometric model of the brace in isometric myodynamia test.

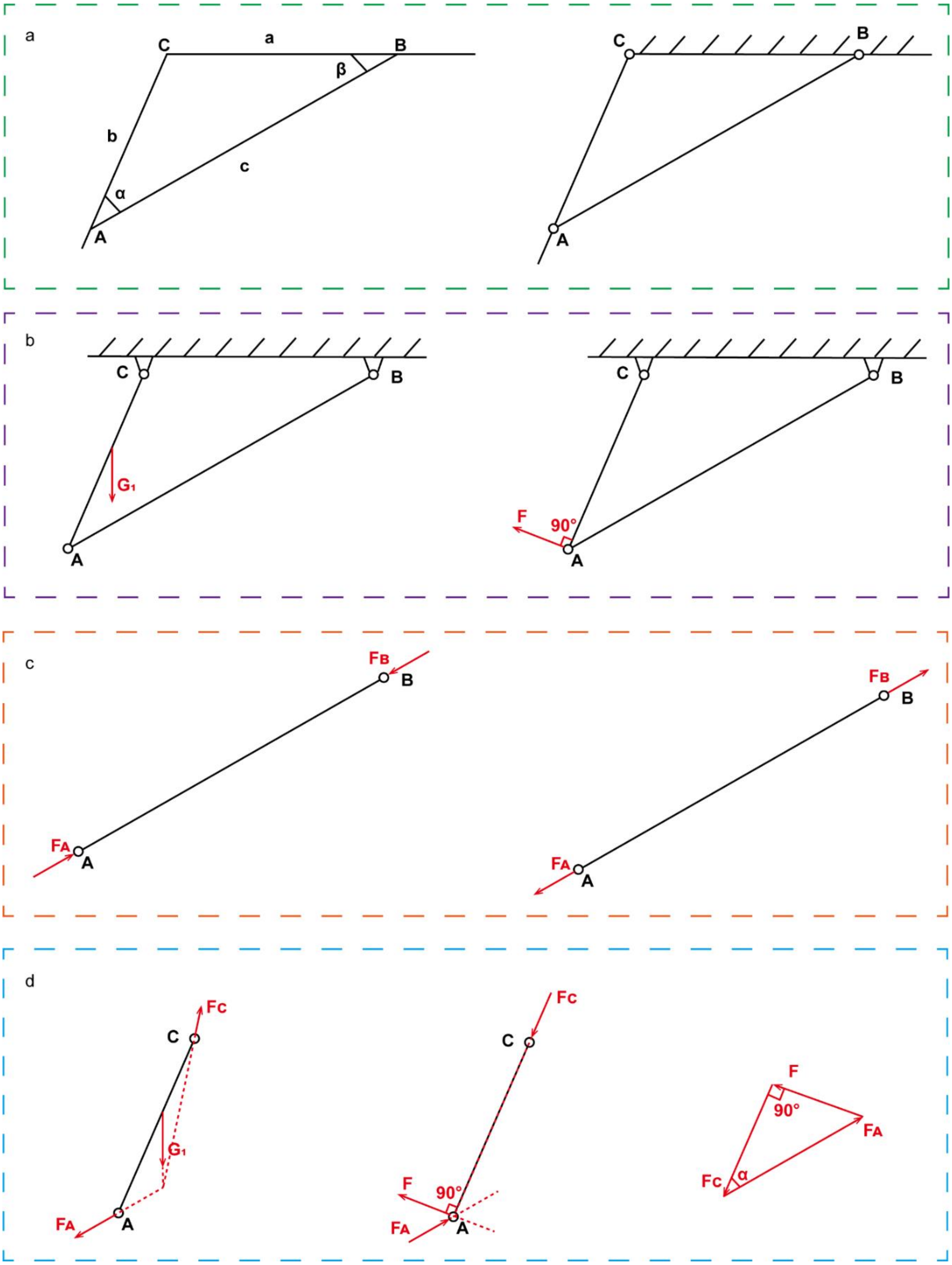

Figure S2. Force analysis of the brace. a) Geometric relationship and simplified model for force deduction **b)** Simplified forces on the brace. **c)** Forces in the AB diastolic bar during isotonic muscle strength test. **d)** Decomposition of gravity and leg force on brace.

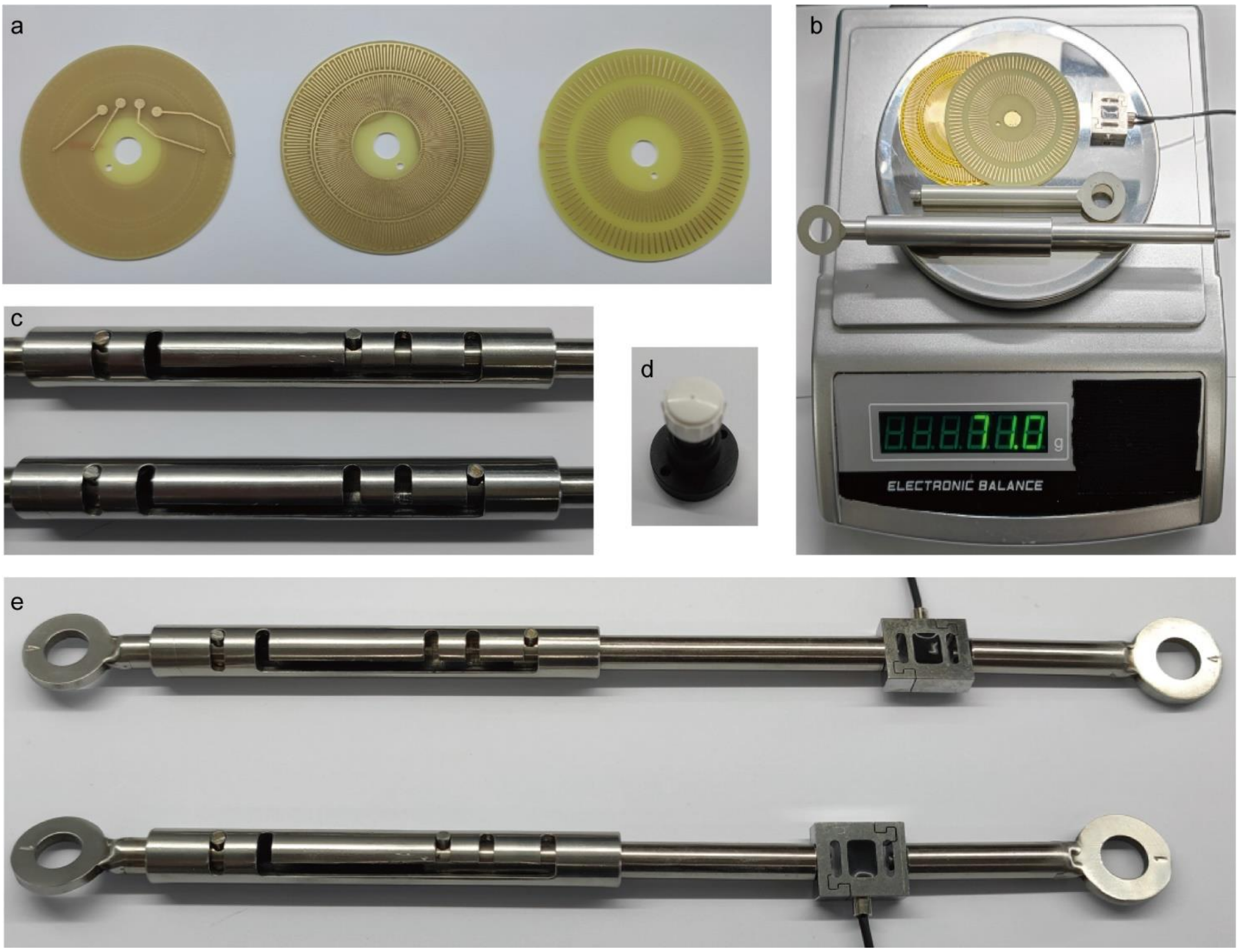

Figure S3. Photos of sensors and telescopic rods. a) Photo of TENG angle sensors. **b)** Sketch of telescopic rod and force transducer. Weight of the attachable modules. Here, the materials of the telescopic rods are aluminum alloy. **c)** Partial photo of the telescopic rods. Here, the materials of the telescopic rods are stainless steel. **d)** Photo of quick release buckle, which is fixated on brace. **e)** Photo of telescopic rods and force gauges. The length represents angle 30 ° and 60 °.

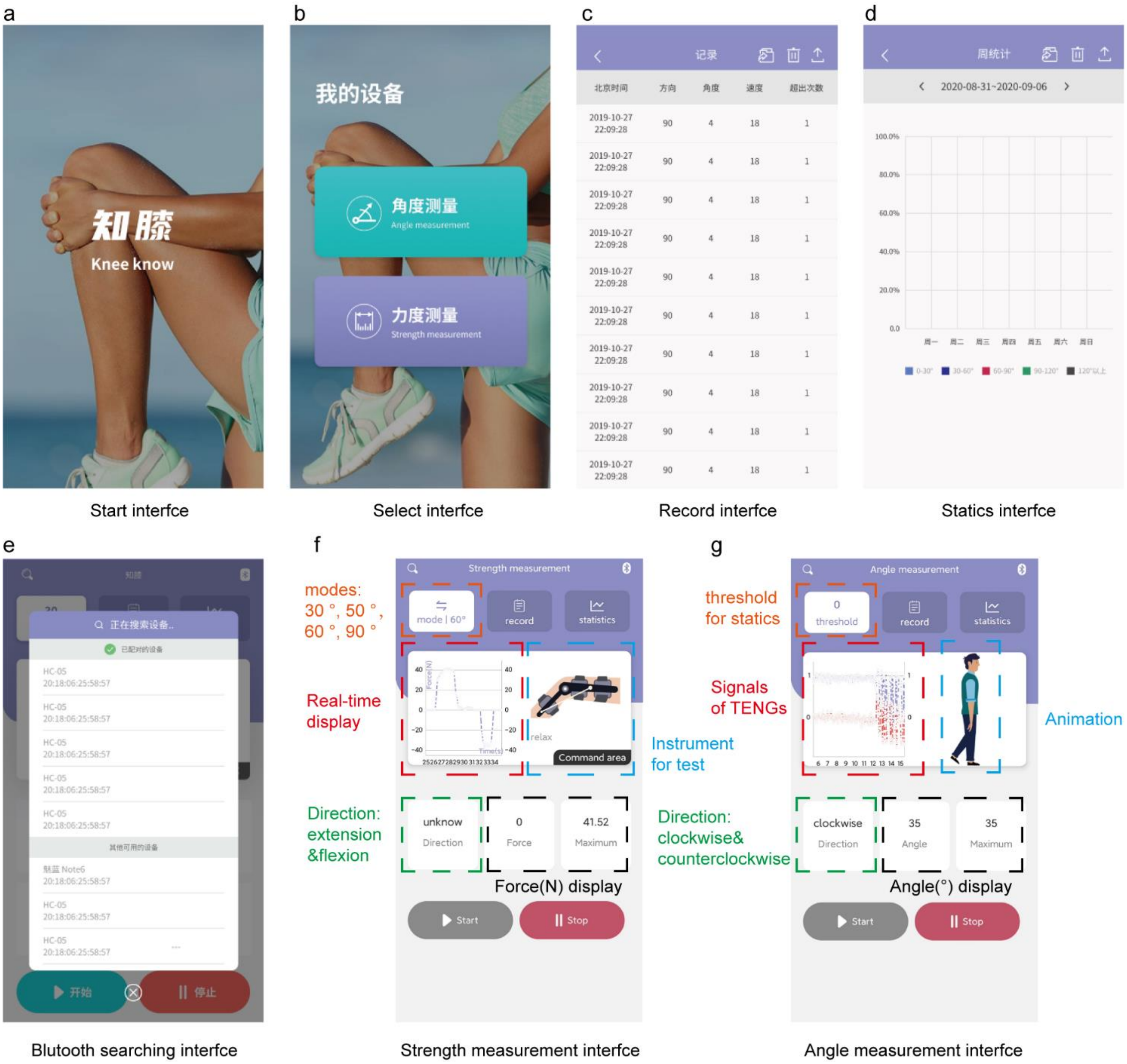

Figure S4. Interfaces of the "Knee Know" APP. Considering that most of the participants' native language is Chinese, the application interface is mainly in Chinese, with some bilingual interfaces. **a)** Start interface. **b)** The selection screen includes an angle measurement portal and a strength measurement portal. **c)** Record interface, taking the angle module as an example, which includes test time, direction, angle speed, the number of times the threshold is exceeded. Besides, the local file open, data export, and data delete buttons are in the upper right corner. **d)** Statistics interface including weekly and monthly statistics. **e)** Search interface. **f)** Strength measurement interface includes portals of searching device, record interface and statics interface. Other functions are illustrated by words. **g)** Angle measurement interface also includes interfaces mentioned above and other functions are described by words.

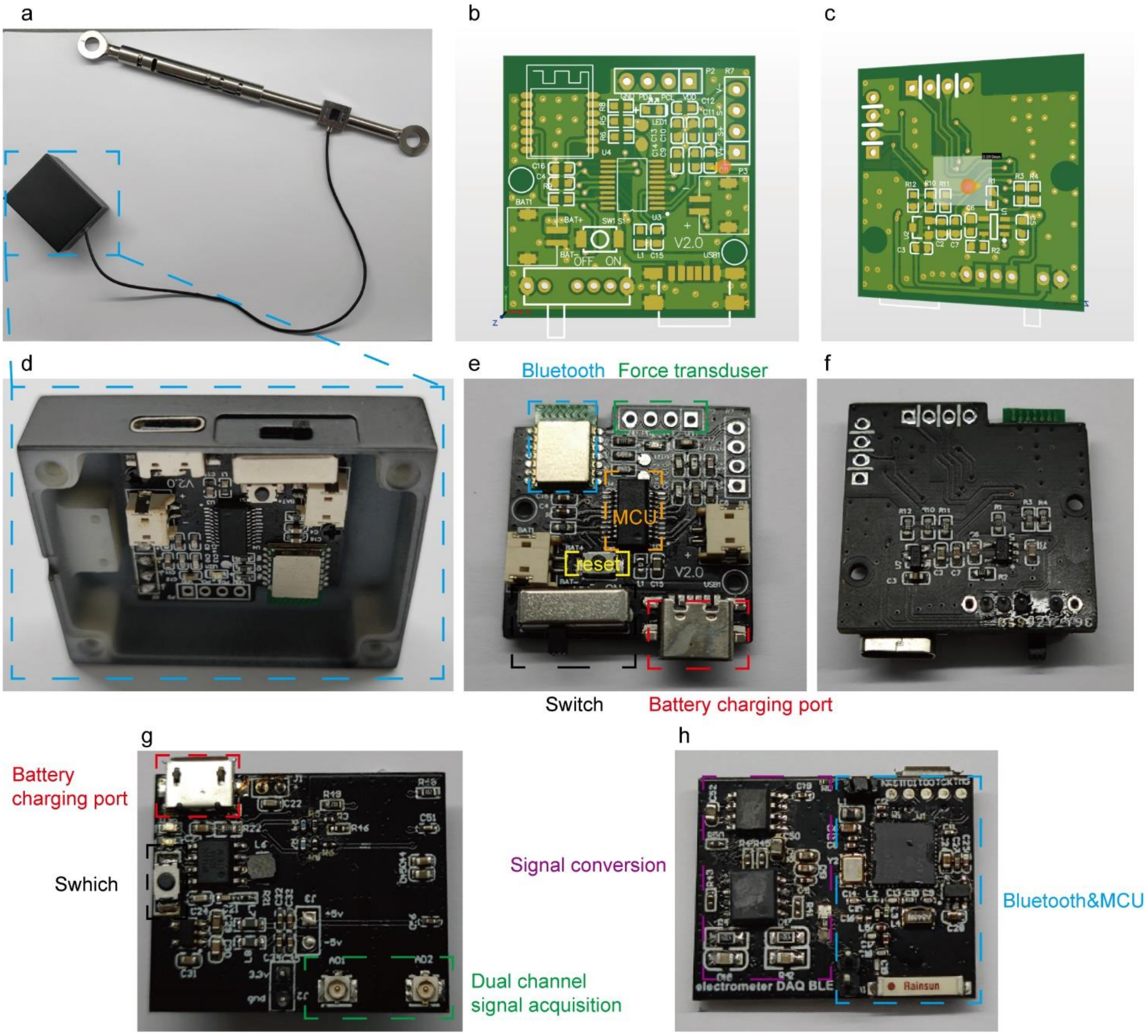

Figure S5. Circuit photos of strength and angle measurement modules. a) Photo of strength measurement module. **b-c)** 3-D views of the strength measurement module circuit in AD **d)** Strength measurement module circuit package picture. **e-f)** Photos of strength measurement module circuit, which is depicted in words. **e)** Photos of angle measurement module circuit.

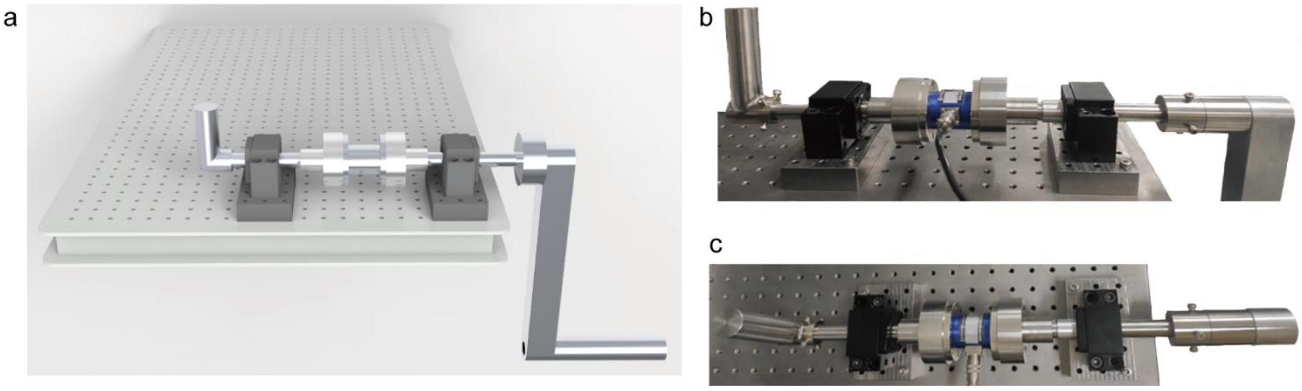

Figure S6. Images of the precise concentric transmission for brace mechanical characterization. a) Picture of the transmission. **b-c)** Photos of the transmission.

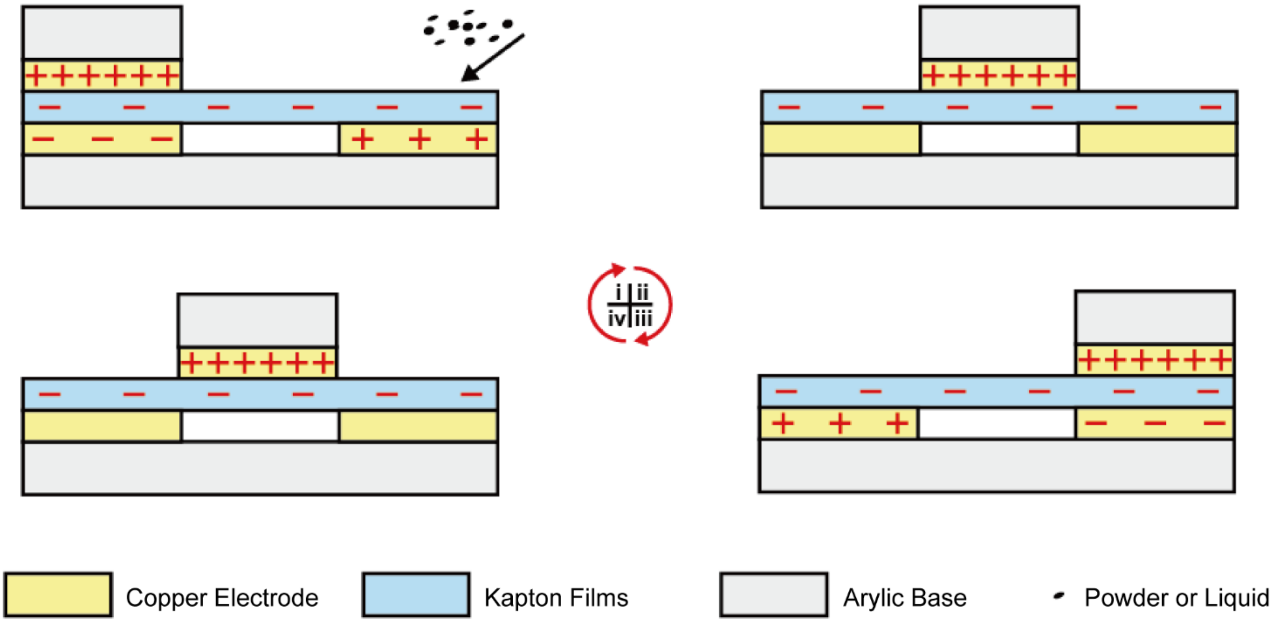

Figure S7. Mechanism diagram of the TENG-based angle sensors.

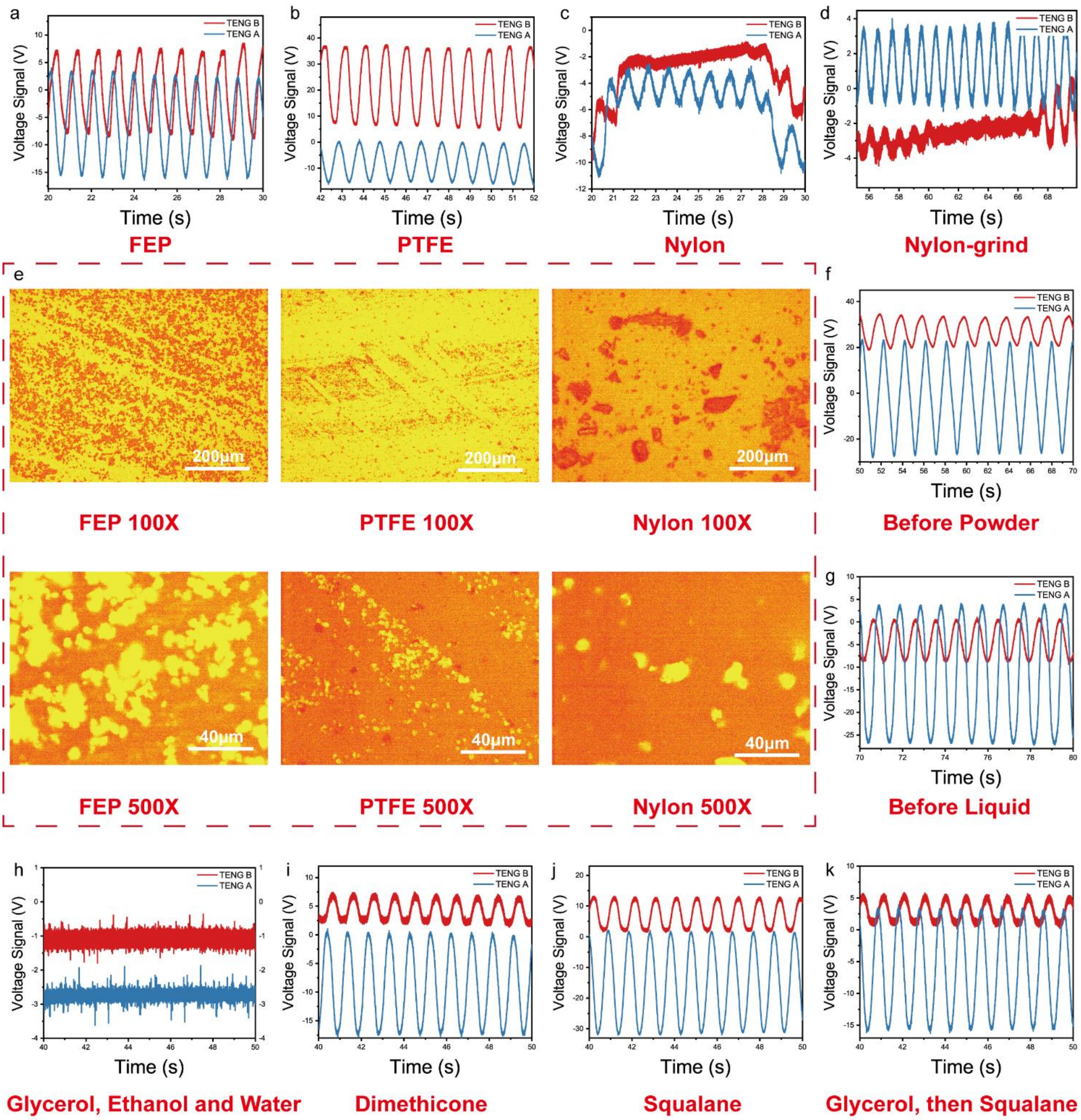

Figure S8. Powder and liquid lubrication for TENG-based angle sensors. To ensure AROM test stability in different environment, lubrication enhancement strategies are set to explore the optimal parameters. **a-d)** Open circuit voltage of two sets of TENGs under FEP, PTFE, Nylon and Nylon-grind lubrication. **e)** Optical microscope photos of FEP, PTFE, and Nylon powders. **f-g)** Open circuit voltage of two sets of TENGs before lubrication **h-k)** Open circuit voltage of two sets of TENGs under glycerol, ethanol, water, dimethicone, and squalene lubrication.

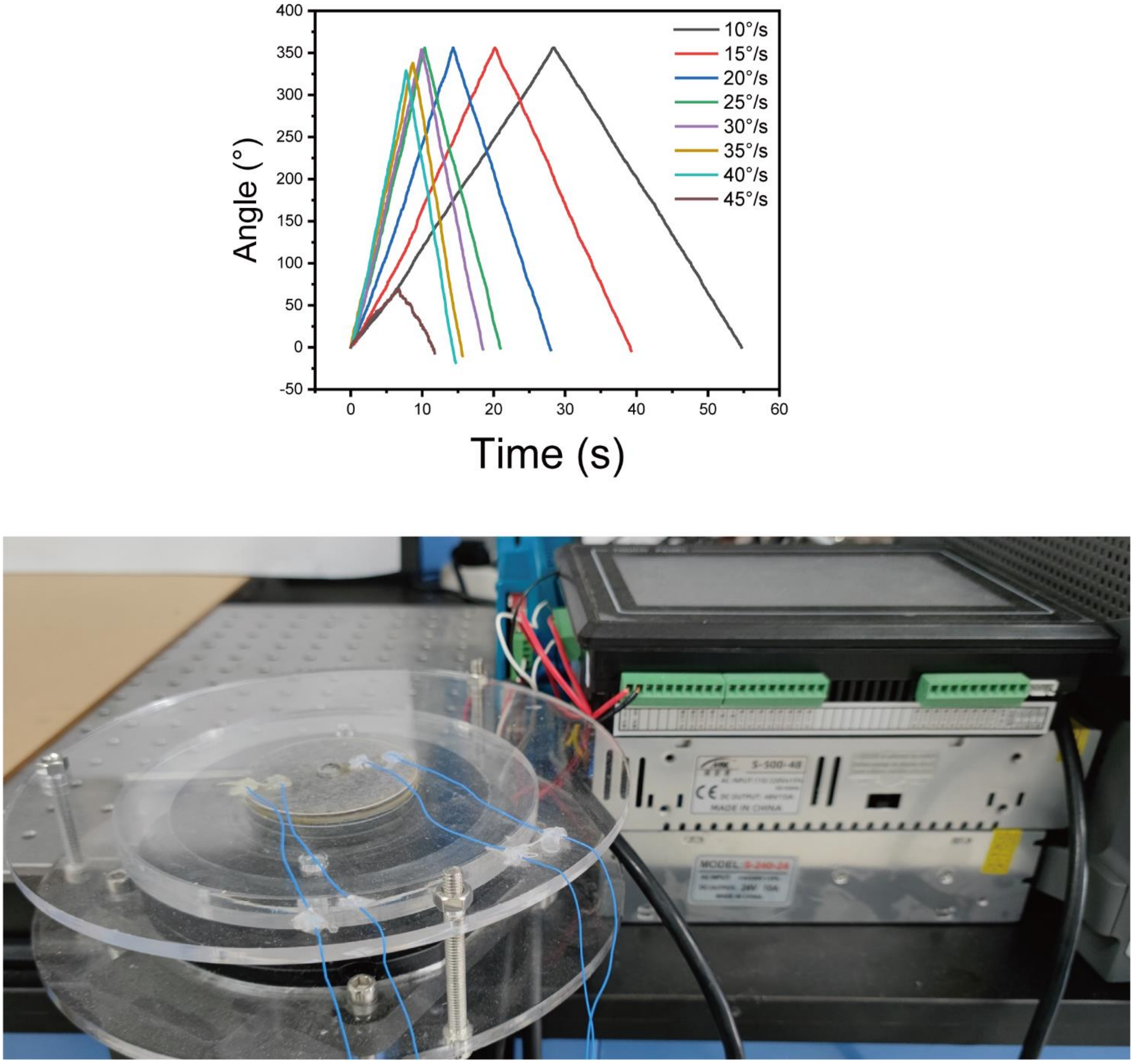

Figure S9. Rotation simulation experiment. Rotating motor is applied to probe the response of active angle sensors at different angular speeds. **a)** Response at speed 10 °/s, 15 °/s, 20 °/s, 25 °/s, 30 °/s, 40 °/s, and 45 °/s. **b)** Photo of the rotating motor and its control system.

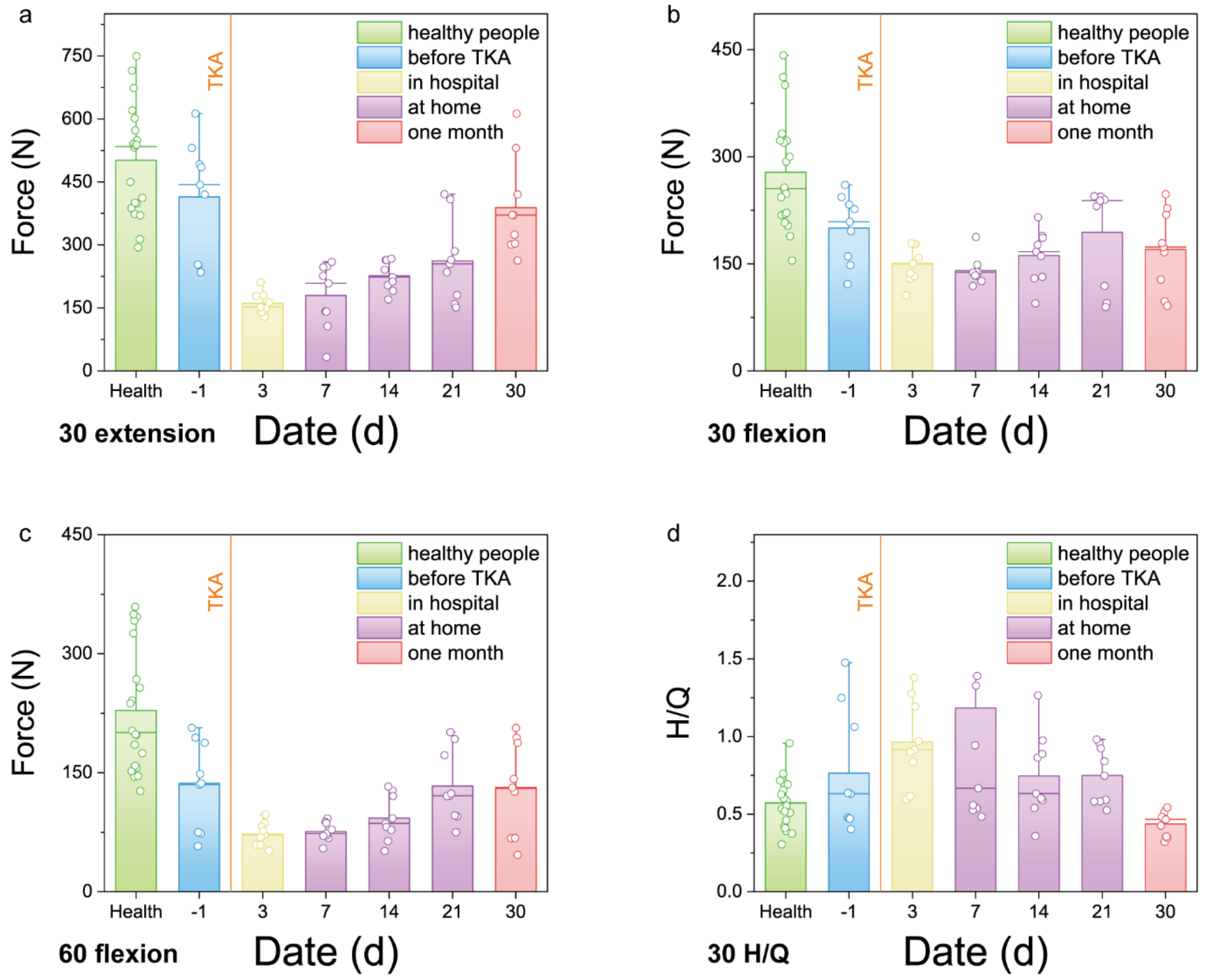

Figure S10. Longitudinal rehabilitation monitoring a group of patients via IMT. a) 30 extension data. **b)** 30 flexion data. **c)** 60 flexion data. **d)** 30 H/Q data.

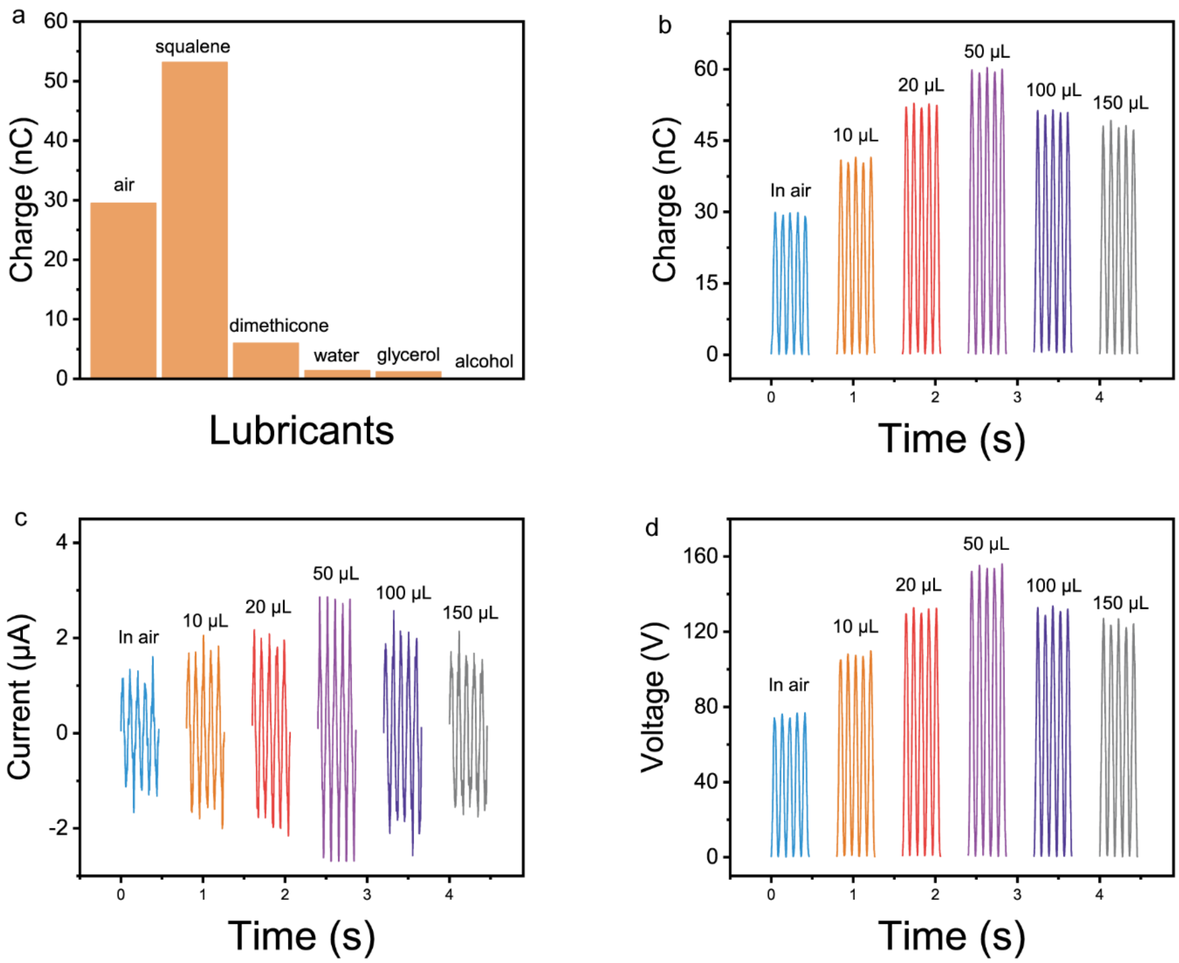

Figure S11. TENG output under different lubricant situations. a) Transferred charge when different lubricants are introduced (20 μL; 10 N; 30 °/s). **b)** Transferred charge, **c)** 60 short-circuit current, and **d)** open-circuit voltage under squalene lubrication (different volume; 10 N; 30 °/s).

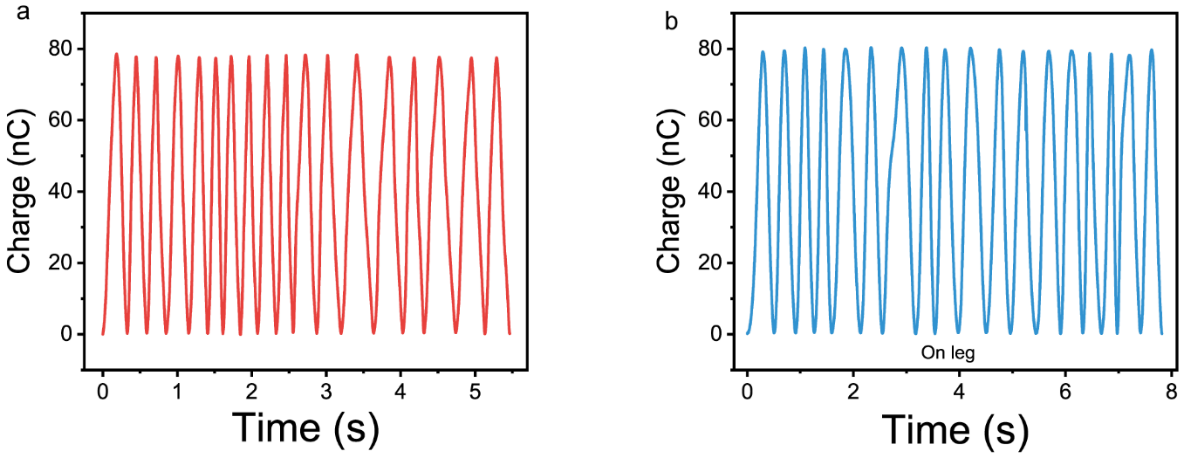

Figure S12. The output performance of the TENG angle sensor in two places: including a) on the experiment workbench and b) on the human leg (embedded in brace).

Captions for the Supporting videos

Video S1. Isometric myodynamia measurement. The test is realized by the bed and force data is visualized on the phone via "Knee Know" APP.

Video S2. Active range of motion measurement. the angle of brace is detected the TENG-based angle sensor when the participant is lying on the bed.
